# Assessment of Current Practices and Feasibility of Routine Screening for Critical Congenital Heart Defects — Georgia, 2012

**Published:** 2013-04-19

**Authors:** Pamela Clark, Johanna Pringle, Regina M. Simeone, Suzanne M. Gilboa, Margaret A. Honein, Matthew Oster, Elizabeth C. Ailes

**Affiliations:** Georgia Dept of Public Health; Oak Ridge Institute for Science and Education; Div of Birth Defects and Developmental Disabilities, National Center on Birth Defects and Developmental Disabilities; EIS Officer, CDC

In September 2011, the U.S. Secretary of Health and Human Services recommended that critical congenital heart defects (CCHD) be added to the Recommended Uniform Screening Panel (RUSP) for newborns. Anecdotal reports in early 2012 suggested that some Georgia hospitals had begun screening for CCHD using pulse oximetry. To better understand the prevalence of routine CCHD screening, specific practices among screening hospitals, and barriers to screening among all birthing hospitals in the state, CDC and the Georgia Department of Public Health (DPH) conducted two surveys of Georgia hospitals in June 2012. Eleven pulse oximetry screenings at five hospitals also were observed to estimate screening time. The initial survey was sent to 89 birthing hospitals, among which 71 (80%) responded; 22 (31%) reported currently screening for CCHD and 20 (28%) planned to start in 2012. Barriers to screening included lack of a clear follow-up protocol for positive screening tests, uncertainty about reporting screening results to public health organizations, and cost concerns. Sixteen (73%) currently screening hospitals responded to the second survey. Only one third of screening hospitals followed the CCHD screening protocol endorsed by the American Academy of Pediatrics; the remaining hospitals screened at different times or had different criteria for a positive screen. Screening time averaged 10 minutes per newborn. In the absence of a state mandate, routine screening has begun in many Georgia hospitals. Use of a standardized screening protocol for CCHD could reduce current variation in screening practices among Georgia hospitals. Working agreements between hospitals also are needed to ensure access to echocardiography and follow-up of newborns with possible CCHD.

Congenital heart defects are associated with approximately eight births per 1,000 (1); approximately 25% of these defects are CCHD and require surgery or cardiac catheterization at age <1 year (2). Many CCHDs are detected prenatally or during physical examination after birth, but some infants with CCHD are discharged home without a diagnosis, putting them at risk for severe disability or death (3). In 2010, the Secretary’s Advisory Committee on Heritable Disorders in Newborns and Children recommended that CCHD be added to the RUSP, and in September 2011, the Secretary accepted the committee’s recommendation.* Currently, screening for CCHD is accomplished through pulse oximetry, a noninvasive test used to detect hypoxemia, which typically is present for the seven CCHD that are the primary targets of pulse oximetry screening (3). The predictive values and sensitivity of pulse oximetry screening varies based on the screening protocol that is used (e.g., timing of screening after birth or number of extremities measured) (4). Despite this federal recommendation to include CCHD on the RUSP, implementation is a state decision. Although universal CCHD screening currently is not mandated in Georgia, anecdotal reports in early 2012 indicated the practice had begun in some birthing hospitals.

DPH requested assistance from CDC to assess the current practices and feasibility of routine screening for CCHD in Georgia. In June 2012, CDC and DPH distributed a survey about CCHD screening practices using pulse oximetry to nurse managers at all the 89 Georgia birthing hospitals. Hospitals could complete the survey online, via fax, or by telephone. The 71 hospitals that completed the initial survey represented 80% of all birthing hospitals in Georgia and accounted for 87% of all live births in the state in 2011 (5). CDC and DPH distributed a follow-up online survey about specific screening procedures to the 22 hospitals that reported in the initial survey that they were currently screening for CCHD using pulse oximetry; 16 (73%) responded. From the 22 hospitals currently screening, a convenience sample of five were selected, at which CDC and DPH staff members observed five screening demonstrations and six actual screenings. Assessment of five screenings included quantification of transport time to and from the nursery, and six did not because other procedures (e.g., metabolic screening) were conducted during these same nursery visits. Two-sided Fisher’s exact tests (significance level of 0.05) were used to assess the statistical significance of differences in the prevalence of hospital characteristics by screening status.

Of the 71 hospitals that responded to the initial survey, 22 (31%) reported currently screening for CCHD using pulse oximetry in their well-baby nursery (11 began in 2010 or 2011, nine in 2012, and two did not indicate when they started); 34 (48%) had plans to start (20 by the end of 2012 and 14 at other times); 14 (20%) had no plans to start; and one did not know of plans to start. No differences by hospital screening status were noted in the number of live births in 2011, availability of echocardiography onsite for infants, or in the availability of pediatric cardiologists for follow-up of babies with CCHD (Table). Several barriers to CCHD screening were reported more frequently among nonscreening hospitals (Table). Overall, 46 (65%) hospitals reported that they could perform echocardiography on-site. For follow-up of patients with suspected CCHD, 32 (45%) had to transfer patients. The median driving distance to a transfer hospital was 54 miles (range: 0–211 miles).

Among 16 (73%) of the 22 screening hospitals that responded to the follow-up survey, five (31%) reported following the CCHD screening protocol† endorsed by the American Academy of Pediatrics, the American College of Cardiology, and the American Heart Association (6). The remaining hospitals either screened at different times or used different criteria for a positive screen. No hospital reported providing written documentation to parents about the screening. Among the 16 hospitals, 12 did not know how often to send screening data to DPH and 11 did not know what types of screening data, such as true and false positives and negatives, could be sent to DPH. Four of the 16 screening hospitals had identified one or more infants with a CCHD through screening. Thirteen of the hospitals neither hired extra staff nor added extra staff hours to accommodate CCHD screening, and three did not respond to the question. The average time to conduct and document the 11 observed screens was 10 minutes per screen (range: 3–15 minutes).

CDC recommended that 1) guidance be provided to hospitals on the type of data to report to DPH, and the frequency of reporting; 2) an educational webinar be developed for hospitals on signs and symptoms of CCHD and the pulse oximetry screening protocol endorsed by the American Academy of Pediatrics; 3) educational materials that hospitals can provide to parents about CCHD screening be developed and disseminated; and 4) working agreements between hospitals be established to ensure access to echocardiography and follow-up for all newborns with possible CCHD.

## Editorial Note

In Georgia, a state without mandated CCHD screening, at least 42 birthing hospitals, accounting for 60% of births in the state (5), are conducting routine CCHD screening in their well-baby nursery (20) or planned to start (22) by the end of 2012. Frequently cited barriers to CCHD screening include the lack of a clear protocol for follow-up for positive screening results, uncertainty about how to report results to Georgia DPH, and cost concerns. In addition, many hospitals are unable to perform echocardiography on-site or have to transfer patients for follow-up of suspected CCHD. Even among hospitals already screening, screening protocols and practices varied.

What is already known on this topic?

In September 2011, the U.S. Secretary of Health and Human Services recommended that critical congenital heart defects (CCHD) be added to the Recommended Uniform Screening Panel for newborns. Universal screening for CCHD using pulse oximetry is not mandated in Georgia, but anecdotal reports in early 2012 suggested screening had begun in some birthing hospitals.

What is added by this report?

Among 71 of 89 Georgia birthing hospitals that responded to the initial survey, 42 (59%) reported currently (22) or planning to start (20) screening for CCHD using pulse oximetry by the end of 2012. Barriers to screening in some hospitals and variation in screening practices remain. Nearly one third of hospitals are unable to perform echocardiography for infants on-site in their facility and almost half need to transfer newborns with possible CCHD to another facility for follow-up and diagnosis.

What are the implications for public health practice?

Implementation of routine screening for CCHD in the absence of a state mandate has led to variation in screening protocols. Use of a standard screening protocol and educational programs might alleviate these differences. Hospitals need recommendations as to what screening data to collect and report. Working agreements between hospitals are needed to ensure access to echocardiography and follow-up of newborns with suspected CCHD.

Published reports from other states are limited. A survey of Wisconsin hospitals found similar results to this assessment; approximately 25% of Wisconsin hospitals had voluntarily begun screening. Barriers to screening included lack of access to echocardiography, long transfer hospital distances, and variation in screening procedures and protocols (7). The average screening time of 10 minutes per newborn from this assessment is greater than previous estimates of 2–3.5 minutes (8,9). Despite the added potential burden of approximately 274 hours per year devoted to CCHD screening for the typical Georgia birthing hospital (based on a mean of 1,642 births among hospitals currently screening or planning to begin screening by the end of 2012), none of the hospitals that responded to the survey added staff or hours to accommodate screening.

The findings in this report are subject to at least three limitations. First, the survey response rates were 80% to the initial survey and 73% to the second survey. Nonresponders might have had different CCHD screening experiences from responders; if so, these results might not be applicable to all birthing hospitals in Georgia. Second, screening practices were reported by the nurse manager who filled out the survey and might not reflect those of all nurses in a given facility. Finally, the numbers of hospitals conducting CCHD screening and the specific screening procedures used are likely to change over time, so the results of this assessment might not reflect Georgia hospitals’ current screening practices.

The findings from this assessment of CCHD screening practices in Georgia might be useful to other states. Routine screening has voluntarily begun in many Georgia hospitals, although screening practices vary and not all hospitals are able to provide appropriate follow-up for infants with possible CCHD. Georgia hospitals need guidance on a standardized screening protocol for CCHD. Working agreements also need to be created between hospitals to ensure access to echocardiography and follow-up of newborns with possible CCHD in Georgia hospitals.

## Figures and Tables

**TABLE:** Characteristics of 71 Georgia birthing hospitals currently screening for CCHD, planning to start soon, or with no plans to start screening,* as of June 2012

	Currently screening (n = 22)		Planned to start screening in 2012 (n = 20)		Plan to start screening at other times (n = 14)		No plans to start screening or unknown* (n = 15)		
					
Characteristic	No.	(%)§	No.	(%)§	No.	(%)§	No.	(%)§	p-value†
**No. of births (2011)**									
Mean	1,837		2,062		999		1,424		
(Range)	(175–5,500)		(111–1,752)		(200–3,300)		(165–3,238)		
Median	1,475		800		812.5		1,164		0. 427
**Total**	**40,411**		**39,185**		**13,992**		**21,353**		
**How hospital records or plans to record pulse oximetry screening results**									0.093
EMR only	15	(68)	11	(55)	9	(64)	12	(80)	
Paper only	4	(18)	6	(30)	5	(36)	0	(0)	
Both EMR and paper	2	(9)	3	(15)	0	(0)	0	(0)	
Missing	1	(5)	0	(0)	0	(0)	3	(20)	
**Hospital has facilities to perform diagnostic echocardiography on-site for infants**									0.844
Yes	15	(68)	14	(70)	8	(57)	9	(60)	
No one available	6	(27)	6	(30)	6	(43)	5	(33)	
Don’t know if available	1	(5)	0	(0)	0	(0)	1	(7)	
**Availability of pediatric cardiologists for follow-up and diagnosis of babies born with CCHD**									0.067
Specialists are on-site at hospital	0	(0)	0	(0)	1	(7)	2	(13)	
Consultants with a specialty group see patients on-site at hospital	10	(45)	8	(40)	2	(14)	4	(27)	
Echocardiography are reviewed remotely; hospital transfer patients if further cardiac care is needed	3	(14)	2	(10)	0	(0)	4	(27)	
Hospital transfers patients out to another facility	9	(41)	10	(50)	9	(64)	4	(27)	
Hospital does not have pediatric cardiologist available at all	0	(0)	0	(0)	1	(7)	1	(7)	
Other	0	(0)	0	(0)	1	(7)	0	(0)	
**Barriers to screening**									
No clear plan for follow-up of positive results	5	(23)	6	(30)	8	(57)	5	(33)	0.211
Unsure of how to report results	4	(18)	5	(25)	6	(43)	7	(47)	0.200
Concerned about reimbursement for cost of screening (but no need for new staff or equipment)	7	(32)	8	(40)	4	(29)	2	(13)	0.402
Need to purchase new equipment to carry out the screening	2	(9)	7	(35)	7	(50)	5	(33)	**0.043**
No state mandate for screening	1	(5)	1	(5)	2	(14)	7	(47)	**0.003**
Waiting to hear about experiences of other hospitals	0	(0)	3	(15)	6	(43)	2	(13)	**0.004**
Believe number of false positives will be too high	1	(5)	1	(5)	2	(14)	4	(27)	0.168
Believe CCHD infants will be picked up through other mechanisms	0	(0)	0	(0)	0	(0)	3	(20)	**0.014**
Need to hire new staff to carry out the screening	0	(0)	0	(0)	1	(7)	1	(7)	0.240
Other									
Developing screening policies and guidance and educating staff about them	3	(14)	7	(35)	2	(14)	2	(13)	0.302
Physician support	2	(9)	4	(20)	2	(14)	3	(20)	0.727
Staff time	1	(5)	3	(15)	0	(0)	0	(0)	0.263
More evidence about pulse oximetry screening needed	0	(0)	1	(5)	0	(0)	2	(13)	0.214
Documentation of results	2	(9)	0	(0)	0	(0)	0	(0)	0.333
No barriers	9	(41)	2	(10)	1	(7)	2	(13)	**0.040**

**Abbreviations:** EMR = electronic medical record; CCHD = critical congenital heart defects.

* Includes responses from the one hospital that did not know its CCHD screening status.

† Fisher’s exact test, comparison of all nonmissing responses or Kruskal-Wallis test for difference in median number of live births. Significant p-values (<0.05) are in bold.

§ Percentages might not sum to 100% because of rounding.

## Footnotes

* Available at http://www.hrsa.gov/advisorycommittees/mchbadvisory/heritabledisorders/recommendations/correspondence/cyanoticheartsecre09212011.pdf.

† Available at http://www.cdc.gov/ncbddd/pediatricgenetics/pulse.html.

## References

[1] Reller MD, Strickland MJ, Riehle-Colarusso T, Mahle WT, Correa A (2008). Prevalence of congenital heart defects in metropolitan Atlanta, 1998–2005. J Pediatr.

[2] Hoffman JI, Kaplan S (2002). The incidence of congenital heart disease. J Am Coll Cardiol.

[3] Mahle WT, Newburger JW, Matherne GP (2009). Role of pulse oximetry in examining newborns for congenital heart disease: a scientific statement from the American Heart Association and American Academy of Pediatrics. Circulation.

[4] Thangaratinam S, Brown K, Zamora J, Khan KS, Ewer AK (2012). Pulse oximetry screening for critical congenital heart defects in asymptomatic newborn babies: a systematic review and meta-analysis. Lancet.

[5] Hamilton BE, Martin JA, Ventura SJ (2012). Births: preliminary data for 2011. Table 6. Births by race and Hispanic origin of mother: United States and each state and territory, preliminary 2011. Nat Vital Stat Rep.

[6] Kemper AR, Mahle WT, Martin GR (2011). Strategies for implementing screening for critical congenital heart disease. Pediatrics.

[7] Beissel DJ, Goetz EM, Hokanson JS (2012). Pulse oximetry screening in Wisconsin. Congenit Heart Dis.

[8] Walsh W (2011). Evaluation of pulse oximetry screening in Middle Tennessee: cases for consideration before universal screening. J Perinatol.

[9] Bradshaw EA, Cuzzi S, Kiernan SC, Nagel N, Becker JA, Martin GR (2012). Feasibility of implementing pulse oximetry screening for congenital heart disease in a community hospital. J Perinatol.

